# A dataset of Curie and Néel temperatures auto-generated with ChemDataExtractor and the Snowball algorithm

**DOI:** 10.1038/s41597-025-06244-6

**Published:** 2025-12-12

**Authors:** Charles R. Kelly, Jacqueline M. Cole

**Affiliations:** 1 Cavendish Laboratory, University of Cambridge, J. J. Thomson Avenue, Cambridge, CB3 0HE UK; 2 STFC Rutherford Appleton Laboratory, Harwell Science and Innovation Campus, Didcot, Oxfordshire, OX11 0QX UK

**Keywords:** Magnetic properties and materials, Magnetic materials, Ferromagnetism

## Abstract

An auto-generated dataset of Curie and Néel temperatures is presented, containing 56,037 records extracted from 108,181 published scientific articles. Each record contains the extracted chemical entity and associated extracted temperature. The dataset was auto-generated by mining text from the papers using the ‘chemistry-aware’ natural-language-processing toolkit, ChemDataExtractor 2.2.2, in conjunction with the Snowball v2 parser, which has been adapted to extract Curie and Néel temperatures from scientific text. This dataset is the first of its kind to be generated using the Snowball v2 parser, with its evaluation yielding a precision of 72% and a recall of 61%. The public availability of this dataset will aid in the design, prediction and analysis of magnetic materials.

## Background & Summary

The ‘trial-and-error’ method for materials design and discovery cannot meet the pace of demand for new materials. Thus, data-driven approaches are progressively replacing such methods^1^. The foundation for realising experimental data for such approaches involves using data-scraping tools such as the ‘chemistry-aware’ natural-language-processing (NLP) software, ChemDataExtractor^2–5^; this toolkit can produce large and accurate datasets of chemical information about the structural characteristics and properties of materials by mining the scientific literature. Examples of such datasets exist for a variety of application areas including photovoltaics^5^, optics^6^, thermoelectrics^7^, batteries^8^, optoelectronics^9^, semiconductors^10^ and stress-strain engineeering^11^. The exploitation of these datasets and the significant advances in machine learning (ML) methods allows for a significant acceleration of materials discovery and a corresponding reduction in the current 20-year average “molecule-to-market” time frame that is required to deliver a new material in a product using traditional methods^12,13^.

There have also been extensive computational efforts to accelerate materials design and discovery. In 2011, the Materials Genome Initiative^14,15^ was deployed to aid the discovery and design of new materials using big data. Since then, many projects and roadmaps have arisen such as the Materials Project^16^, the materials-by-design roadmap^17^, Automatic Flow for Materials Discovery^18–20^ and NOvel MAterials Discovery (NOMAD)^21^.

Despite such efforts, there remains a lack of large domain-specific computational datasets that have been experimentally verified. This is partly due to the difficulty in gathering a vast amount of labelled experimental data into a single corpus even though they exist; for example in the scientific literature, albeit such data lie fragmented across myriad papers in multiple journals of many publishers.

The ChemDataExtractor^2–5^ toolkit was designed to provide a solution to the challenge of gathering large amounts of data on materials-property relationships. By employing NLP and ML methods, ChemDataExtractor can search for and extract chemical entities and specific properties from scientific literature that have been produced by a variety of publishers. This gives the user easy access to a substantial amount of material-property relationship data that they can tailor to their specific research needs and collate the data in an easy-to-use organised dataset.

**Fig. 1:**
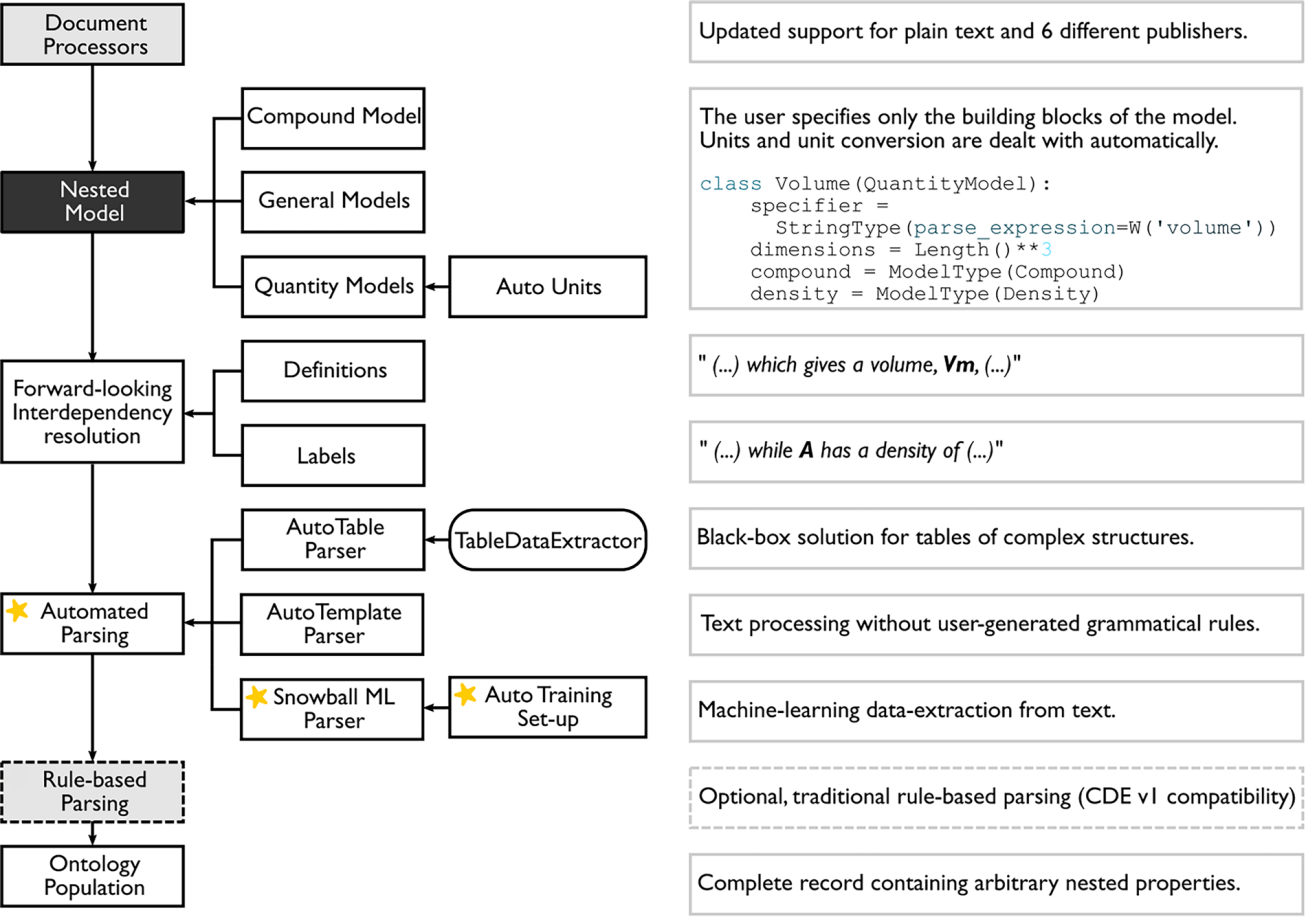
Complete pipeline of chemical data extraction with Snowball v2 integration using ChemDataExtractor 2.0. Yellow stars indicate necessary user interaction, and the boxes with dashed borders are optional steps. Taken from ref. ^23^ Copyright 2021 American Chemical Society.

The original ChemDataExtractor toolkit^2^ has undergone major development since its first release in response to user needs, with version 2.0^3^, 2.1^4^ and 2.2^5^ presenting key new features. In addition, its semi-supervised ML parser, Snowball v1^22^, has been massively overhauled; thereby, the capability of Snowball v2^23^ vastly outshines that of its predecessor. Figure 1 shows the inner workflow of the current version of ChemDataExtractor (v2.2.2) with the Snowball v2 parser integrated into its implementation, as highlighted by stars.

Snowball v1 formed an explicit part of the earlier ChemDataExtractor v1.3 pipeline that was used to auto-generate a dataset of magnetic materials and their Curie and Néel phase-transition temperatures^22^. Given these major new implementations of ChemDataExtractor and Snowball, we undertook a fresh construction of this magnetic dataset on magnetic materials. For reference, Curie and Néel phase-transition temperatures are the temperatures at which paramagnetic materials order into ferromagnetic and anti-ferromagnetic materials, respectively.

This dataset is the first large-scale materials dataset to be generated using the Snowball v2 parser, integrated within ChemDataExtractor v2.2.2. Its semi-supervised ML algorithm had been pre-trained on over 1000 sentences^23^. Compared with the Snowball v1 parser, Snowball v2 requires considerably less setup and input from the user for custom property extraction. The Snowball v2 parser also achieved a higher performance threshold than the custom modular-style parsers that were developed for ChemDataExtractor using its previous method of data extraction.

The performance of this new dataset for magnetic materials was evaluated to have a precision of 72% and a recall of 61%. A conventional parser was also developed using the built-in functions of ChemDataExtractor, whose comparative performance was analysed on the same evaluation set as per the Snowball v2 parser. It transpired that Snowball v2 performed significantly better with precision, scoring 11% higher than when the conventional ChemDataExtractor parser was used. In contrast, the recall from using Snowball v2 was 14% lower than when the conventional ChemDataExtractor parser was employed. However, the conventional parser uses phrase parsing that required weeks of manual fine-tuning to develop. In comparison, the Snowball v2 parser comes packaged as a pre-trained generic parser requiring mere minutes of setup.

## Methods

### Data scraping

A corpus was created by scraping 108,181 journal articles from Elsevier and the Royal Society of Chemistry (RSC) publishers, using simple web-scraping scripts that make use of the Elsevier Application Programming Interface (API) and the Selenium web driver for python. These scraping scripts require a query input from the user, which the scraper then uses to filter out irrelevant articles, only returning articles that contain the keywords that the user requires. Search queries of “Curie Temperature” and “Néel Temperature” were submitted to both publishers. In the case of Elsevier, this search first returns all articles from all available journals; its web driver then visits the publisher’s website using the query to search for the relevant articles and sends a request to download them to the user on their local machine. Elsevier returns articles in an.xml format while the RSC returns articles in a .html format.

### Developing the conventional parsers using in-built functions of ChemDataExtractor

ChemDataExtractor uses sentence-level parsing for data mining and provides a series of easy-to-use base classes which can be easily adapted by the user to extract specific chemical information that is tailored to their research. The parsers consist of several “keyword terms” that ChemDataExtractor uses to find the sentences that contain Curie and Néel temperatures within the text. These keyword terms are: “Curie Temperature”, “Néel Temperature”, “T_c_”, “T_N_”, “T_Curie_”, and “T_Néel_”.

The parser was initially developed by inspecting scientific articles that had been scraped in the corpus gathering process, to see which phrases commonly surround text or symbols that denote Curie or Néel temperatures. These phrases were written into the parser for ChemDataExtractor to use for its search, examples of which are: “was determined to be”, “remains at”, and “observed as”. The parser was then tested on these papers to evaluate if the material-property relationship was extracted. This process was iterated over roughly 1800 papers to boost the performance of the parser as much as possible.

**Fig. 2:**
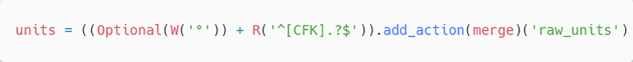
A parsing grammar for the units of temperature.

The parser presented is split into five main sections: units, value, prefix, scenarios, and suffix. This takes advantage of several built-in functions and tags within ChemDataExtractor for sentence parsing. Since we are only looking for one property, temperature, the valid units are limited to Celsius, Fahrenheit, and Kelvin. Figure 2 shows the parsing grammar that filters for just these units.

‘W’ is a case-sensitive tag and ‘R’ is a regular expression (Regex) rule tag. The ‘add_action(merge)’ is used to group these two components together, and finally the ‘raw_units’ tag is added at its end. In this grammar, the Regex rule will filter for the temperature abbreviations F, C, or K, with an optional superscripted “°” before it, thereby covering for all possible units of temperature.

**Fig. 3:**
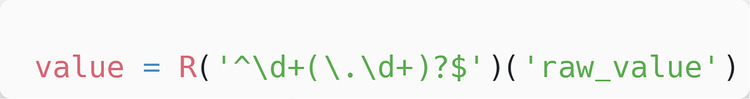
A Regex expression for finding a numerical value.

The next stage of the parser provides a grammar for obtaining the numerical value of the temperature. Again, this is achieved using a simple Regex rule, as shown in Fig. 3, with the tag ‘raw_value’ being added to its end.

**Fig. 4:**
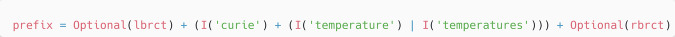
An example grammar in the prefix list of the parser.

The prefix of the parser contains a list of phrases that are commonly found at the start of the sentence containing these temperature characteristics. An example is included in Fig. 4. Here the ‘I’ tag is used to define a case-insensitive word. The ‘lbrct’ and ‘rbrct’ tags are used to define left and right brackets within the text.

**Fig. 5:**
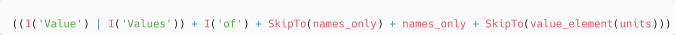
An example grammar in the scenarios list of the parser.

The scenarios section of the parser contains a phrase list of common words that are typically found between the prefix and the value. An example of a scenario is shown in Fig. 5. The parser takes advantage of several built-in functions within ChemDataExtractor for sentence parsing, which have been demonstrated here. The ‘SkipTo’ function is used to skip over all tokens in a sentence until a certain condition is met. In the cases shown in Fig. 5, those conditions are the ‘names_only’ function, and the ‘value_element(units)’ function.

The suffix of the parser is a list of characters that often appear immediately before a value. This would typically be an equals sign, which ChemDataExtractor accounts for internally. However, there are other rather more ambiguous possibilities for which this list accounts; e.g., “≈” and “~”.

The parser was designed in this modular fashion to enable the easy addition of phrases to each section, to ensure an easy iterative improvement cycle. When ChemDataExtractor is run on an article, it checks for all combinations of all the prefixes, scenarios, and suffixes within the text, thus covering a large basis of possible combinations. There are, however, some drawbacks. ChemDataExtractor parsers can only be developed up to a certain point. During parser development, scenarios were found where the conventional parser for ChemDataExtractor would fail in a way that could not be addressed with parsing rules. Examples of such issues are:

- Properties formatted in such a way that no space separates the prefix from the measured value, e.g. “Tc=30 K”, compared to “Tc = 30 K”.
- Sentences formatted in such a way that multiple properties and compounds are listed in the same sentence, sometimes without units for every value. For example: “Compounds x, y, and z were found to have Curie temperatures of x1 °C, y1 °C and z1 °C” or “Compounds x, y, and z were found to have Curie Temperatures of x1, y1, and z1 °C”.

### Snowball v2 parser

Snowball v2 is a semi-supervised ML algorithm^24^ that we have developed into a generic sentence parser (Snowball v2)^23^ for ChemDataExtractor. Its pre-trained nature allows for material-property relationship extraction with little-to-no development or setup for the end user, nor the requirement of any extra training. The parser comes pre-trained on over 1000 sentences that contain chemical-property relationships about semiconductor bandgap data^23^ upon which Snowball v2 was originally tested. The pre-trained Snowball v2 configuration addresses the previously listed intrinsic limitations of the conventional ChemDataExtractor parsers. In particular, Snowball v2 provides a vast improvement in recall for articles in which information is formatted in such a way that the conventional ChemDataExtractor parsers fail.

Another huge advantage of Snowball v2 is that there is no need for any type of extensive and iterative parser development that is described in the previous section for conventional parsing. Snowball v2 has an almost “plug-and-play” implementation within ChemDataExtractor, making the user experience nearly seamless.

**Fig. 6:**
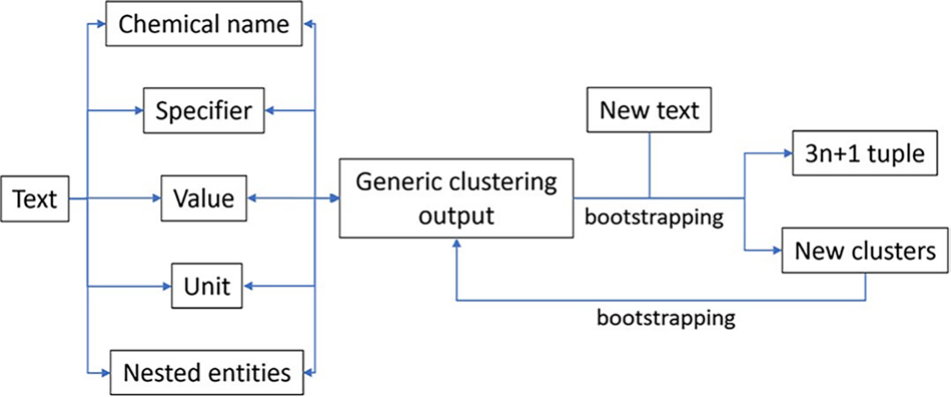
Operational structure of the Snowball 2.0 algorithm. Boxes contain textual data; single-ended arrows indicate computing processes where the tail box provides input for the head box; and double-ended arrows indicate reversibility in the clustering stage. Taken from ref. ^23^ Copyright American Chemical Society.

The generic Snowball algorithm employs bootstrapping, and Snowball v2 provides improvements to this over its predecessor. Bootstrapping is a form of feedback within the algorithm that it uses to learn, so that it can become better at parsing. Snowball v2 includes changes to this part of the algorithm, enabling new information to be learnt faster and more efficiently. Figure 6 outlines the internal pipeline of Snowball v2.

**Fig. 7:**
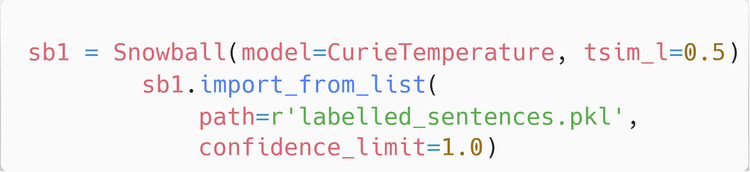
Initialising a Snowball v2 instance.

In common with a conventional ChemDataExtractor parser, the Snowball v2 parser is provided with a target query, with which it can search within sentences for property extraction. As with the conventional ChemDataExtractor parser that was tailored for this study (see previous section), the query phrases used were: “Curie Temperature”, “Néel Temperature”, “T_c_”, “T_N_”, “T_Curie_”, and “T_Néel_”. Once the target query has been set, a Snowball instance has to be initialised so that it can be used as a parser, as illustrated in Fig. 7.

There are several variables involved in initialising a Snowball v2 instance:

- The ‘model’ variable points to the model that contains the query specifier; in this case, this is the Curie Temperature model.
- The ‘tsim_l’ is the lower similarity threshold, which in this case is set to 50%.
- The ‘path’ variable points to the file containing the labelled sentences upon which the model has been trained; this file is provided with the Snowball v2 source code.
- The ‘confidence_limit variable’ is set to 100% in this case, which ensures that Snowball v2 only imports phrases which are guaranteed to be correct.

**Fig. 8:**
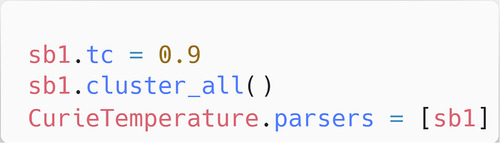
Clustering the imported Snowball phrases into the model.

Figure 8 demonstrates how phrases are clustered into the model; these concern the final steps that are required to prepare the Snowball v2 as a parser for ChemDataExtractor. First, the ‘tc’ variable was set to 90% in this study, which is the minimum confidence threshold for cluster generation. Finally, the phrases can be clustered into a model using ‘cluster_all()’, and the Curie Temperature model for ChemDataExtractor can then have the clustered Snowball v2 model assigned as its parser. This is the last user-interaction step required to prepare the parser, at which point the ChemDataExtractor can be pointed to the article directory and information extraction can commence.

### Material-property relationship extraction

Before information can be extracted by ChemDataExtractor, it first needs to be converted into plain text for ChemDataExtractor to read. To do this, ChemDataExtractor contains several built-in readers for converting a document into machine-readable text. These readers take advantage of structure tags that are built into the syntax structure of common document formats such as HTML and XML. For example, the <p> tag defines the location of a paragraph in HTML. The reader can identify this and then note that all text contained within the <p> tag constitutes a paragraph. Other common tags include <header> for a paragraph heading, and <table> for defining a table. The plain text can then be passed to the bespoke natural-language-processing (NLP) pipeline of ChemDataExtractor for material-property relationship extraction which is made up of five key main stages: tokenisation, word clustering, part-of-speech (POS) tagging, chemical named-entity recognition (CNER), and phrase parsing. Each of these five stages has been tailored for mining scientific literature in the chemistry domain. For example:

The tokenisation stage incorporates the Punkt algorithm by Kiss and Strunk^25^ to identify abbreviations (Fig., Et. Al., Ref., etc) in sentences to assist in correctly identifying sentence boundaries.

The word-clustering stage is combined with unsupervised learning to improve the POS tagging and CNER components of the pipeline. Due to a lack of annotated text collections in the chemistry domain that can be used for supervised learning, ChemDataExtractor uses the Brown clustering method to provide a substitute for the hierarchal clustering of words based on their frequency and the context in which they occur^26^.

Whilst most publicly available POS taggers are trained on a corpus of newspaper articles, the POS system that ChemDataExtractor implements has been trained on a corpus of newspaper articles and MEDLINE abstracts by Tsuroka *et al*.^27^ to improve its performance in the chemistry domain, which is further complemented by word-cluster features.

The CNER component in ChemDataExtractor v1^2^ uses a word list compiled by the Jochem chemical dictionary^28^ as a look up for chemical names, in conjunction with rule-based algorithms that achieve CNER on text in the chemistry domain where conventional NLP systems often fail. Extended capability of the CNER component was introduced in ChemDataExtractor v2.1^4^, with the integration of a language model built on the Bidirectional Encoder Representations from Transformers (BERT) architecture^29^. The language model employed is a fine-tuned SciBERT model for CNER. The original SciBERT model was pretrained on the Semantic Scholar Corpus^30^, which consists of a large group of papers across scientific disciplines, resulting in a model more apt to scientific domain tasks such as CNER, over the original BERT model. The work by Isazawa and Cole^4^ includes the fine-tuning of SciBERT on the CHEMDNER^31^ and Matscholar^32,33^ datasets, embedding knowledge of both organic and inorganic materials within the model.

Phrase parsing in ChemDataExtractor uses specialised grammars that have been designed for chemical information to work around a problem caused by the differences in language used within scientific literature compared to everyday language. Phrases are parsed to grammars as a list of tags generated by the CNER and POS systems which contain nested rules that decide how a sequence of tokens can be converted into a tree model which represents the structure of a phrase.

Once material-property relationships have been identified by ChemDataExtractor, they need to be structured in a useful way. To achieve this, the custom python packages e2e_workflow^34^ CDEDatabase^35^ were used in conjunction. Both e2e_workflow and CDEDatabase are custom python packages developed for use with ChemDataExtractor. The former provides a full end-to-end workflow for parsing a corpus to ChemDataExtractor in a parallel-computing environment. Once ChemDataExtractor has performed the data extraction, e2e_workflow passes the extracted records into CDEDatabase where they are encoded into a structured Java Standard Object Notation (JSON) dataset. JSON is a well-known, easy-to-use file format. The user can create simple scripts to read JSON in a variety of programming languages to easily access the dataset. A further breakdown of how the records within the JSON dataset are structured is given in the Data Records section.

## Data Cleaning

Whilst undertaking manual evaluations of the performance of parsers during their early stages of development, it was noted that some data records about compounds contained negative values with the units of Kelvin, leading to false positives. These values typically stemmed from cases where “Paramagnetic Curie Temperatures” was mentioned within the originating text. These material-property relationships were not being targeted by our parsers for extraction but were still being classified as Curie temperatures due to the similarity in wording. As such, a data post-processing script was written to scrub the dataset of any record that met both of the following criteria:

- A raw value that is less than 0.
- A raw unit of Kelvin.

This post-processing step naturally resulted in a diminution of the total number of data records in the final dataset. However, it greatly increased the precision of the dataset.

## Data Records

**Fig. 9:**
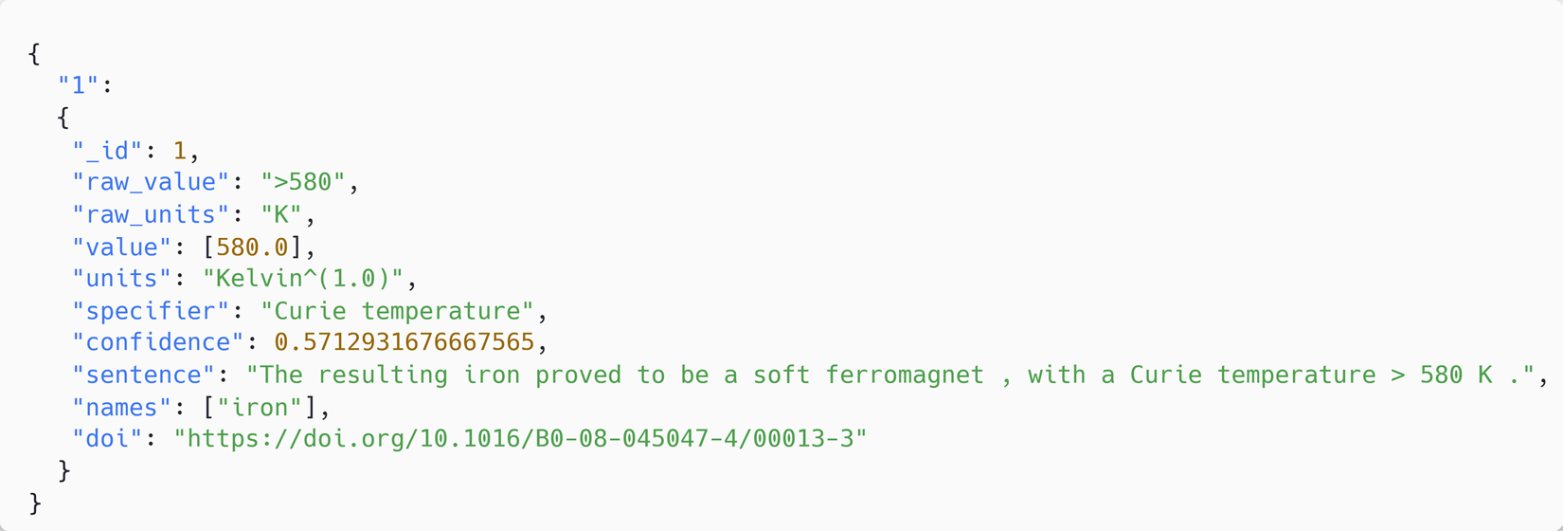
An extract of a full record from the dataset.

The study herein resulted in a dataset of 56,037 records of Curie and Néel temperatures which has been made publicly available on Figshare^36^. These records have been structured in both JSON and CSV formats for ease-of-use, with the dataset keys outlined in Table 1. Each record starts with a numerical id notating its position within the dataset. The raw value and raw unit’s fields contain the values and associated units as they are found within the source. Following this, the value and units fields contain values and units normalised to a standard form. The raw specifier and containing sentence are provided for each record showing where the material-property relationship was extracted from the source material in an unedited form. We also provide the confidence score that the Snowball v2 parser assigns to each extracted sentence in a normalised form. Finally, the names field provides a list of compounds that were extracted by the parsers presented herein, and the doi field provides the DOI number of the paper from which the record was extracted. An extract of a full record from the dataset is given in Fig. 9. There are currently no further plans to expand or update this dataset.

**Table 1:** A description of the records within the Curie-Néel temperature dataset.

dataset Key	Description	Data Type
_id	Numeric position within dataset	Integer
raw_value	Raw extracted value without normalisation	String Type
raw_units	Raw extracted units without normalisation	String Type
value	Normalised extracted values	List, Float
units	Normalised extracted units	String Type
specifier	The target string located by the parser within the source	String Type
confidence	Confidence value assigned to extracted sentence by Snowball v2 parser	Float
sentence	Original sentence where relationship was found	String Type
Names	List of extracted compounds associated with extracted values	List, String Type
doi	URL of the article in which the record was extracted from	String Type

## Technical Validation

To evaluate the performance of the dataset, metrics of precision and recall were determined using the definitions of Eqs. 1 and 2:

1 $${Precison}=\frac{{TP}}{{TP}+{FP}}$$

2 $${Recall}=\frac{{TP}}{{TP}+{FN}}$$

where ${TP}$ denotes a true positive, ${FP}$ denotes a false positive and ${FN}$ denotes a false negative.

The evaluation process involved choosing, at random, 100 published articles that had been scraped from RSC journals and manually evaluating the extracted records from those published articles. Not all of these articles contained relevant data about magnetic materials. So, to ensure a fair evaluation, this process was repeated until it was possible to accrue more than 100 papers whose manual evaluation had produced at least 1 true positive, false positive, or false negative record for each paper using Snowball v2 as the parser. Given that the process involves 100 papers per repeating cycle, the total number of evaluated papers with at least 1 TP, FP, or FN record found, turned out to be 115. This entire process was then repeated from the outset using published articles scraped from Elsevier instead. Once more, published articles were chosen at random in sets of 100, except that the process was stopped when 115 manually evaluated articles had returned at least 1 TP, FP, or FN record, so that an even comparison between the two publishers was possible.

If the compound name, value, and unit in a data record all matched, the extracted record is said to be a true positive. If any combination of the compound name, value, or unit in the data record were found to be incorrect, the extracted record is said to be a false positive. If the published article contained a Curie or Néel temperature with an associated unit and compound within the text, but this information was not extracted by the parser, this is said to be a false negative. It can therefore be said that precision is a measure of accuracy of the extracted records that make up the dataset, and recall is a measure of the parser’s ability to successfully extract targeted data records within the text of the published articles.

As part of this technical validation, a like-to-like comparison was made between the data-extraction performance of the new Snowball v2 parser and that of the conventional ChemDataExtractor2 parser. Both parsers used the same evaluation set in this performance testing for precision and recall, *i.e*. the set of 230 randomly selected papers (115 from the RSC and 115 from Elsevier).

**Fig. 10:**
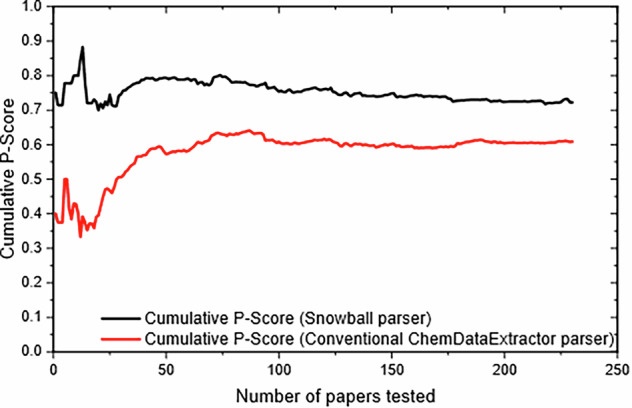
A performance comparison in precision (P), between the Snowball v2 parser and the conventional ChemDataExtractor parser, shows Cumulative P-Scores against Number of Papers evaluated.

Figure 10 describes the cumulative P-Score for both the Snowball v2 parser (shown in black) and the conventional CDE parser (shown in red) as each test progresses. The precision naturally varies a lot initially because the first few papers sampled will not be representative of the entire set. Only after ~75 papers have been sampled does the precision stabilise to a nearly constant value, *i.e*. the representative (average) precision. The results from the tests on both parsers revealed an 11% greater precision for the Snowball v2 parser.

**Fig. 11:**
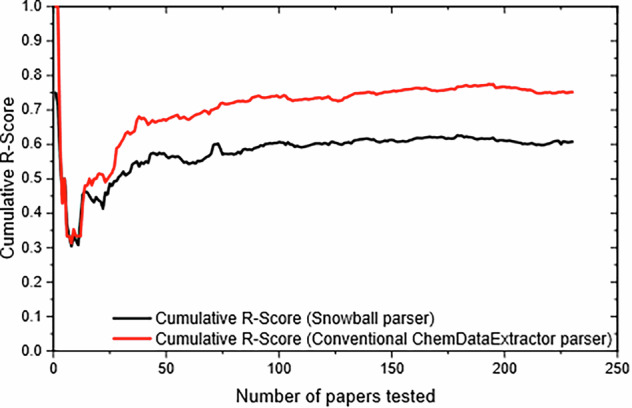
A performance comparison in recall (R), between the Snowball v2 parser and the conventional ChemDataExtractor parser, shows Cumulative R-Scores against Number of Papers evaluated.

Figure 11 describes the corresponding cumulative R-Score from the same performance tests on both the Snowball v2 parser (shown in black) and the conventional ChemDataExtractor parser (shown in red). The Snowball v2 parser was shown to perform 14% less well than the conventional ChemDataExtractor parser in terms of recall, based on the evaluation set of 230 randomly selected papers.

In summary, the generic Snowball v2 parser displayed a significant improvement in precision over the conventional ChemDataExtractor parser, and yet a significant reduction in recall. It should be noted, however, that the conventional ChemDataExtractor parser took weeks of development, manually adding and tweaking various phrases, to afford the precision and recall numbers that are presented herein. This is in stark contrast to the situation for the Snowball v2 parser, which comes pre-trained and takes just minutes to setup and return comparable results. This study presents the first auto-generated dataset that has employed the generic Snowball v2 parser, and this parser was used in a purely out-of-the-box fashion.

Finally, we present a comparison of this study with previous work. While this study showcases a fresh look on the work in by Court and Cole^22^ using the vastly improved and overhauled ChemDataExtractor v2.2.2 and Snowball v2, it is important to note some differences between the studies. In the study conducted by Court and Cole, Snowball v1 was used as a “top-up” to complement the dataset generated by the conventional parsing methods in ChemDataExtractor v1, with Snowball v1 generating just 1,350 of the total 39,822 records in their dataset. Conversely, we used Snowball v2 as our sole parser for dataset generation. ChemDataExtractor v2.2.2^5^ introduces a completely different architecture to that of ChemDataExtractor v1, with the inclusion of a novel NLP pipeline, tokenization, and a model concept for extracting material-property relationships, as opposed to strict rule-based parsing used in ChemDataExtractor v1. The vast differences in the pipelines make a direct algorithmic comparison between the two studies impossible; however, we have included a numerical analysis of the two datasets.

The study by Court and Cole (2018) achieved an estimated precision of 73% across 39,822 records from a corpus of 68,078 papers. Court and Cole scraped their corpus from Elsevier and Springer papers, whereas the dataset presented herein was scraped from Elsevier and Royal Society of Chemistry (RSC) papers. Our estimated precision was very similar, at an estimated 72%, across 56,037 records from a corpus of 108,181 papers. The reported recall for Court and Cole’s study was 56%, which is 5% lower than our 61%, though it should be noted that the analysis for this Court and Cole’s recall was performed with a manual evaluation over 50 papers, whereas their precision was derived from a manual evaluation over 200. As the precision and recall estimate for both datasets involve the manual evaluation of a sample of records, it is impossible to give the total number of true positive, false positive, and false negative records for either dataset. To address this, we have provided metrics of both datasets in Table 2.

**Table 2:** A direct comparison between the dataset in this study and that presented by Court and Cole^22^.

Dataset	Court and Cole (2018)	This study
Precision	73%	72%
Recall	56%	61%
Corpus Size	68,078	108,181
Publishers scraped	Elsevier, Springer	Elsevier, RSC
Total records	39,822	56,037
Curie records	28,482	41,647
Néel records	11,340	14,390
N.O unique compounds	17,097	18,819
Common compounds	3,740	

**Fig. 12:**
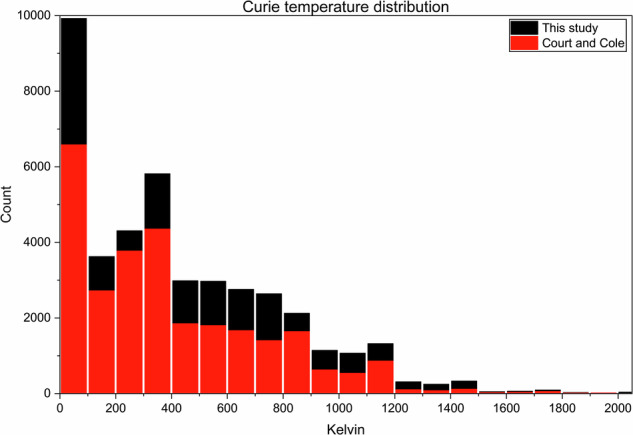
Distribution of Curie temperatures in the dataset presented in this study (black) and the dataset presented by Court and Cole (red).

**Fig. 13:**
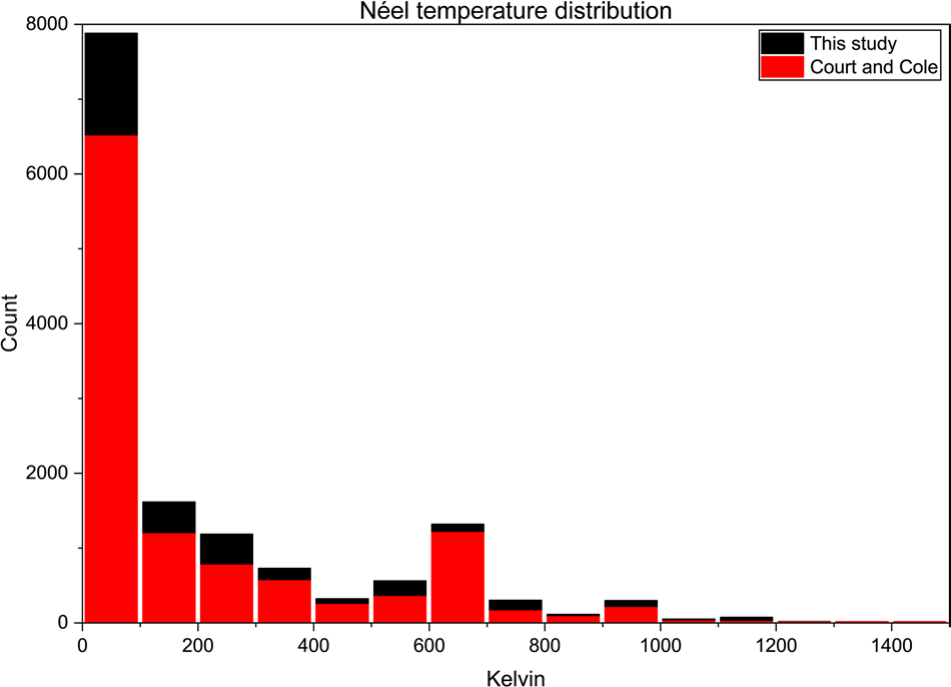
Distribution of Néel temperatures in the dataset presented in this study (black) and the dataset presented by Court and Cole (red).

To further this direct comparison between the two datasets, the distribution of the records containing Curie and Néel temperatures is given in the histograms shown in Figs. 12 and 13. The distributions for both Curie and Néel temperatures in both histograms are very similar, indicating an agreement between the two datasets and providing evidence that this work is a significant advancement on the previous study.

## Data Availability

The dataset for this study is available on Figshare^36^, released under the CC-BY-4.0 license and can be found at 10.6084/m9.figshare.29559686.v2.

## Code availability

The codebase for this study can be found at https://github.com/C-R-Kelly/Curie-Neel-temperature-dataset, released under the CC-BY-4.0 license.

## References

[1] Cai, J., Chu, X., Xu, K., Li, H. & Wei, J. Machine learning-driven new material discovery. *Nanoscale Adv* **2**, 3115–3130 (2020). 36134280 10.1039/d0na00388c PMC9419423

[2] Swain, M. C. & Cole, J. M. ChemDataExtractor: A Toolkit for Automated Extraction of Chemical Information from the Scientific Literature. *J Chem Inf Model* **56**, 1894–1904 (2016). 27669338 10.1021/acs.jcim.6b00207

[3] Mavračić, J., Court, C. J., Isazawa, T., Elliott, S. R. & Cole, J. M. ChemDataExtractor 2.0: Autopopulated Ontologies for Materials Science. *J Chem Inf Model* **61**, 4280–4289 (2021). 34529432 10.1021/acs.jcim.1c00446

[4] Isazawa, T. & Cole, J. M. Single Model for Organic and Inorganic Chemical Named Entity Recognition in ChemDataExtractor. *J Chem Inf Model* **62**, 1207–1213 (2022). 35199519 10.1021/acs.jcim.1c01199 PMC9049593

[5] Isazawa, T. & Cole, J. M. Automated Construction of a Photocatalysis Dataset for Water-Splitting Applications. *Sci Data* **10**, 651 (2023). 37739960 10.1038/s41597-023-02511-6 PMC10517137

[6] Zhao, J. & Cole, J. M. A database of refractive indices and dielectric constants auto-generated using ChemDataExtractor. *Sci Data* **9**, 192 (2022). 35504964 10.1038/s41597-022-01295-5 PMC9065060

[7] Sierepeklis, O. & Cole, J. M. A thermoelectric materials database auto-generated from the scientific literature using ChemDataExtractor. *Sci Data* **9**, 648 (2022). 36272983 10.1038/s41597-022-01752-1 PMC9587980

[8] Huang, S. & Cole, J. M. A database of battery materials auto-generated using ChemDataExtractor. *Sci Data* **7**, 260 (2020). 32764659 10.1038/s41597-020-00602-2 PMC7411033

[9] Huang, D. & Cole, J. M. A database of thermally activated delayed fluorescent molecules auto-generated from scientific literature with ChemDataExtractor. *Sci Data* **11**, 80 (2024). 38233439 10.1038/s41597-023-02897-3 PMC10794197

[10] Dong, Q. & Cole, J. M. Auto-generated database of semiconductor band gaps using ChemDataExtractor. *Sci Data* **9**, 193 (2022). 35504897 10.1038/s41597-022-01294-6 PMC9065101

[11] Kumar, P., Kabra, S. & Cole, J. M. A Database of Stress-Strain Properties Auto-generated from the Scientific Literature using ChemDataExtractor. *Sci Data* **11**, 1273 (2024). 39580441 10.1038/s41597-024-03979-6 PMC11585639

[12] Cole, J. M. How the Shape of Chemical Data Can Enable Data-Driven Materials Discovery. *Trends Chem* **3**, 111–119 (2021).

[13] Cole, J. M. A Design-to-Device Pipeline for Data-Driven Materials Discovery. *Acc Chem Res* **53**, 599–610 (2020). 32096410 10.1021/acs.accounts.9b00470

[14] Horton, M. K. *et al*. Accelerated data-driven materials science with the Materials Project. *Nat Mater*, 10.1038/s41563-025-02272-0 (2025). 10.1038/s41563-025-02272-0 40610673

[15] Holdren, J. P. *Materials Genome Initiative for Global Competitiveness*, (2011).

[16] Jain, A. *et al*. Commentary: The Materials Project: A materials genome approach to accelerating materials innovation. *APL Mater* **1** (2013).

[17] Alberi, K. *et al*. The 2019 materials by design roadmap. *J Phys D Appl Phys* **52**, 013001 (2019).

[18] Calderon, C. E. *et al*. The AFLOW standard for high-throughput materials science calculations. *Comput Mater Sci* **108**, 233–238 (2015).

[19] Curtarolo, S. *et al*. AFLOWLIB.ORG: A distributed materials properties repository from high-throughput ab initio calculations. *Comput Mater Sci* **58**, 227–235 (2012).

[20] Curtarolo, S. *et al*. AFLOW: An automatic framework for high-throughput materials discovery. *Comput Mater Sci* **58**, 218–226 (2012).

[21] Scheidgen, M. *et al*. NOMAD: A distributed web-based platform for managing materials science research data. *J Open Source Softw* **8**, 5388 (2023).

[22] Court, C. J. & Cole, J. M. Auto-generated materials database of Curie and Néel temperatures via semi-supervised relationship extraction. *Sci Data* **5**, 180111 (2018). 29917013 10.1038/sdata.2018.111 PMC6007086

[23] Dong, Q. & Cole, J. M. Snowball 2.0: Generic Material Data Parser for ChemDataExtractor. *J Chem Inf Model* **63**, 7045–7055 (2023). 37934697 10.1021/acs.jcim.3c01281 PMC10685441

[24] Agichtein, E. & Gravano, L. *Snowball*. in *Proceedings of the fifth ACM conference on Digital libraries* 85–94, 10.1145/336597.336644 (ACM, New York, NY, USA, 2000).

[25] Kiss, T. & Strunk, J. Unsupervised Multilingual Sentence Boundary Detection. *Computational Linguistics* **32**, 485–525 (2006).

[26] Brown, P. F. *et al*. Class-Based n-gram Models of Natural Language. *Computational Linguistics* **18** (1992).

[27] Tsuruoka, Y. *et al*. Developing a Robust Part-of-Speech Tagger for Biomedical Text. in 382–392, 10.1007/11573036_36 (2005).

[28] Hettne, K. M. *et al*. A dictionary to identify small molecules and drugs in free text. *Bioinformatics* **25**, 2983–2991 (2009). 19759196 10.1093/bioinformatics/btp535

[29] Devlin, J., Chang, M.-W., Lee, K. & Toutanova, K. BERT: Pre-training of Deep Bidirectional Transformers for Language Understanding (2018).

[30] Ammar, W. *et al*. Construction of the Literature Graph in Semantic Scholar. in *Proceedings of the 2018 Conference of the North American Chapter of the Association for Computational Linguistics: Human Language Technologies, Volume 3 (Industry Papers)* 84–91, 10.18653/v1/N18-3011 (Association for Computational Linguistics, Stroudsburg, PA, USA, 2018).

[31] Krallinger, M. *et al*. The CHEMDNER corpus of chemicals and drugs and its annotation principles. *J Cheminform* **7**, S2 (2015). 25810773 10.1186/1758-2946-7-S1-S2 PMC4331692

[32] Weston, L. *et al*. Named Entity Recognition and Normalization Applied to Large-Scale Information Extraction from the Materials Science Literature. *J Chem Inf Model* **59**, 3692–3702 (2019). 31361962 10.1021/acs.jcim.9b00470

[33] Tshitoyan, V. *et al*. Unsupervised word embeddings capture latent knowledge from materials science literature. *Nature* **571**, 95–98 (2019). 31270483 10.1038/s41586-019-1335-8

[34] Isazawa, T. e2e_workflow. https://github.com/ti250/e2e_workflow (2023).

[35] Isazawa, T. CDEDatabase. https://github.com/ti250/CDEDatabase (2023).

[36] Kelly, C. R. & Cole, J. M. A database of Curie and Néel temperatures auto-generated from the scientific literature using ChemDataExctractor, 10.6084/m9.figshare.29559686.v2 (2025).

